# Self-Esteem, Individual versus Team Sports

**DOI:** 10.3390/ijerph182412915

**Published:** 2021-12-07

**Authors:** Peter Šagát, Peter Bartik, Anja Lazić, Dragoș Ioan Tohănean, Vasilios Koronas, Ioan Turcu, Damir Knjaz, Cristina Ioana Alexe, Ioana Maria Curițianu

**Affiliations:** 1 Health and Physical Education Department, Prince Sultan University, Riyadh 12435, Saudi Arabia; sagat@psu.edu.sa (P.Š.); pbartik@psu.edu.sa (P.B.); 2 Faculty of Sport and Physical Education, University of Niš, 18000 Niš, Serbia; anja.lazic96@hotmail.com; 3 Faculty of Physical Education and Mountain Sports, Transilvania University of Brasov, 500036 Brasov, Romania; ioan.turcu@unitbv.ro (I.T.); ioana.curitianu@unitbv.ro (I.M.C.); 4 Department Private College Apostolas Pavlos, 119, Kennenti, Panorama, 55535 Thessaloniki, Greece; b.koronas@yahoo.gr; 5 Faculty of Kinesiology, University of Zagreb, 10000 Zagreb, Croatia; damir.knjaz@kif.unizg.hr; 6 Department of Physical Education and Sports Performance, Faculty of Movement, Sport and Health Sciences, Vasile Alecsandri University of Bacau, 157 Marasesti Str., 600115 Bacau, Romania; cristinaioana.alexe@yahoo.com

**Keywords:** self-esteem, individual sport, team sport

## Abstract

On the basis of the integrative concept of self-esteem discussed in sport-related literature, various studies refer to its importance in the context of sports activities. Self-esteem is often understood as a personality trait because it tends to be durable and stable. No accurate description is available regarding the types of sports in which subjects participated. The main purpose of the research was to identify and compare the levels of self-esteem and self-confidence of athletes practicing individual and team sports. The self-esteem and self-confidence levels were measured by the Rosenberg Self-Esteem Scale (Rosenberg 1979) and the Self-Confidence Test (Romek, 2000). All participants were males. Subjects were divided into two categories: 40 for individual sports and 40 for team sports. There were two evaluation periods: P1, the beginning of the preparation period, and P2, the beginning of the competition period. There were statistically significant differences for P1 (*p* < 0.002) and P2 (*p* < 0.003). The differences between the average values of the two periods were 5.8 points and 3.8 points, both favorable to the group of athletes who practiced individual sports. There were significant differences between the individual and team athletes in self-esteem level. Individual athletes presented a higher level of self-esteem.

## 1. Introduction

While watching sports competitions, it can be often seen that athletes are satisfied with victory, unhappy in cases of failure, and excited to compete again regardless of the result. Athletes do not seem to give up. The efforts they show are great, involving sacrifices on various levels, such as being 100% involved in the training, forgetting about themselves and others, not having enough time to spend with family and friends or to socialize, etc. It seems that nothing can stop them from their desire to participate and win sports competitions. Are all athletes the same, regardless of their sport? Do they all see themselves as winners? Self-esteem refers to the personal value that each individual gives himself or, in other words, how much a person likes and appreciates himself or herself. Self-esteem is often understood as a personality trait because it tends to be durable and stable. For this concept, Mullai et al. [1] used synonymous terms, such as pride, self-worth, self-regard, self-respect, and self-integrity. Rosenberg [2] defined self-esteem as a complex cognitive and affective synthesis. He also distinguished between high self-esteem (positive) and low self-esteem (negative). Higher self-esteem is more often associated with success in all areas of life, and low self-esteem is considered to imply depression and anxiety [3,4,5].

On the basis of the comprehensive analysis, some specialists [6,7] consider that self-esteem constitutes a complex psychological construct that concerns four distinct dimensions: basal physics (the body ego); complementary valorization components, including emotional–affective; cognitive; and a broad social dimension. These four sectors seem to be unevenly developed and are considered the main reason why we feel the urge to maximize our potential and successes and, on the other hand, to minimize personal deficits and shortcomings. Self-esteem has as its core the self, which constitutes both the confluence area of the four dimensions mentioned above and the impetus that drives and coordinates the resources of each one. Butler and Gasson [8] drew attention to the fact that self-esteem becomes difficult to conceptually delimit because, in the specialized literature, the term in question has been extended to self-worth, self-belief, self-concept, self-awareness, and self-image. This aspect makes it quite challenging to include all these visions in a complete and universally accepted definition.

On the basis of the integrative concept of self-esteem discussed in sports-related literature, various studies refer to its importance in the context of sports activities. D’Anna et al. [9] conducted a study measuring self-esteem in several categories of athletes (*n* = 78). The general conclusion of this research highlighted the fact that no gender differences were reported, and the subjects with high self-esteem had the ability to perform much better in high-performance sports. The competition itself has the effect of consolidating an increased level of self-esteem as well. Another study [10] covered all sports participation, and the role of gender in predicting self-esteem was assessed. The research pointed out that there were no gender differences concerning self-worth, while it was noted that females reported high self-esteem when participating in several non-competitive sports. No further accurate description is available regarding the types of sports in which the subjects participated. Our study aimed to identify this aspect more precisely, particularly the possible connections between the categories of sports (with close specific characteristics) and the athletes’ levels of self-esteem.

Numerous studies evidence the beneficial impact of exercise (different types over different periods) on self-esteem [11,12,13,14,15]. In a synthesis effort, the main characteristic that refers to individual sports is that the athletes can target personal performance goals without responsibility to the team [16,17]. The athlete can progress at his own pace; he needs independence, self-discipline, perseverance, and self-control. In cases of success, the credit belongs entirely to him, but he can find no other reason but himself when he fails. Many sports need an individual approach, e.g., athletics, swimming, fencing, gymnastics, wrestling, skiing, skating, golf, and tennis. Practicing an individual sport promotes the development of self-respect and the focus of attention, but it can also cause difficulties when competitions are lost. In these situations, there may be a problem related to faith in their own skills.

A vital element of all team sports is team spirit, which includes cooperation, trust, and respect, regardless of personal performance level. Teammates feel success and failure equally. Team sport involves the excellent opportunity to socialize, but at the same time, they might initiate rivalries between teammates, with a negative impact on both individuals and the team. In general, the complexity of these sports is notable because of the higher number of participants. The game responsibilities are most often divided unequally depending on the athlete’s possibilities, skills, and roles. The teammates can substitute themselves, help and support each other, and coordinate their actions in a unitary way toward achieving the team’s goal. Important determinants are effective communication, perseverance, patience, and altruism. Numerous sports require a team approach, e.g., football, basketball, hockey, handball, volleyball, and rugby. Experts assume that significant personality differences exist between the two categories of athletes [18,19,20,21].

It is hypothesized that the level of self-esteem differs regarding the type of sport practiced, especially when discussing individual and team sports and the interactions that occur in sports activities. Our research aimed to identify and compare the self-esteem levels of athletes who practice individual sports and those who practice team sports. The general objective was to verify if and how much the type of sport practiced affects the level of self-esteem.

## 2. Materials and Methods

### 2.1. Participants

Eighty senior-level male Romanian athletes (age = 22 years, SD = 2.31, age range: 18–26 years) who had been practicing the sport for 10–15 years were distributed as follows: 40 athletes—individual sports group (8 alpine skiing, 4 cross-country skiing, 2 biathlon skiing, 15 contact sports, 6 tennis, and 5 athletics) and 40 athletes—team sports group (14 soccer, 6 basketball, 12 handball, 3 volleyball, and 5 hockey). The athletes were members of various sports clubs in Romania that activated the first league. We chose to use only men in this research because the priority of the research was to show the difference between individual and team sports, not to combine gender. The training schedules differed according to the training periods; in the competition period, athletes trained 8 hours/week, and in the preparation period, they trained 16 hours/week. Group sampling was adopted. The participants signed informed consent before data collection. Responses were anonymous to ensure confidentiality.

### 2.2. Materials and Procedures

All athletes included in the research completed a battery of specific scales to investigate the self-esteem level. The paper-and-pencil method was applied to complete the scales. The participants were questioned during two distinct periods: P1, the beginning of the training period, and P2, the beginning of the competition period. Considering the variety of sports and the different competition calendars for specific disciplines, the time difference between P1 and P2 was 2 months (60 days) on average. During the interval between P1 and P2, the athletes focused on performing the training. The participants had different coaches depending on the sports discipline they practiced. There were no planned (intentional) interventions with actions that could affect the increase or decrease of self-esteem. No athletes had sports competitions during this time. Self-esteem was measured by the following specific psychological tools:

*Rosenberg Self-Esteem Scale*, which comprises 10 items of four response variants between Total Disagreement (1 point) and Total Agreement (4 points). Scores are presented in the range of 10–40 points, and when rating the answers, the following classification of values was utilized as a standard: Low self-esteem (10–16 points); Average self-esteem (17–33 points), High self-esteem (34–40 points) [2], α = 0.82—value calculated for the sample of athletes.

*Self-Confidence Test*: includes 30 items, 10 for each of the following three scales: self-confidence (α = 0.91), social courage (α = 0.82), and initiation of social contacts (α = 88). Values of (α) were calculated for the sample of athletes. There are 3 answer variants possible for each item. The value can be 1, 2, or 3 points [22].

### 2.3. Statistical Analysis

Statistical analysis was performed by the IBM SPSS statistics program (IBM Corp., version 26.0; Inc., Chicago, IL, USA). Means ± standard deviation were calculated for each outcome measure. Independent *t*-tests were used to assess differences between the individual and team sports groups for P1 and P2 with significance set at *p* < 0.05; separate dependent-sample *t*-tests were used to assess differences between periods for the individual and collective sports groups with significance set at *p* < 0.05.

## 3. Results

The statistical analysis presented in Table 1 reports significant differences between the two categories of athletes for P1 (*p* < 0.002) and P2 (*p* < 0.003). The differences between the two periods’ average values were 5.8 points and 3.8 points, both favorable to the individual sports athletes. Analysis at the intra-group level reported increased self-esteem in P2 compared with P1 for both groups. The difference regarding the average obtained in the case of individual sports between the two testing periods had a value of 4.4, and in terms of team sports, the value obtained was 6.4. The overall summary shows that the individual sports athletes obtained the highest score (32.20 points) at P2, and the lowest score (22 points) was observed in P1 for athletes practicing team sports. A decrease in the standard deviation was present for P2 in both categories of sports. Individual sports had an SD of 3.15 in P1 and an SD of 1.98 in P2. For team sports, the SD was 3.91 in P1, and the SD was 2.95 in the period of testing P2.

According to the data presented in Table 2, the component “self-confidence” showed statistically significant differences between the two groups of athletes, both for P1 (*p* < 0.010) and P2 (*p* < 0.027). These values of the significance threshold were below 0.01 and 0.05, respectively. Progress of 1 point for individual sports and 1.4 points for team sports was observed between the two testing periods. The “social Courage” component revealed no relevant differences between the two categories of athletes analyzed. The component “initiation of social contacts” presented significant differences between the studied groups. For both the P1 and P2 periods, the group of athletes practicing individual sports presented higher values of the means expressed by *p* < 0.010 and *p* < 0.018, respectively. The progress in scores between P1 and P2 was 0.9 points for individual sports and 1.2 points for team sports.

## 4. Discussion

We conclude that the individual sports category subjects had a higher level of self-esteem than the athletes practicing team sports. This confirms the primary hypothesis of our research. A possible explanation of this fact might be the impact connected with some specific characteristics of the respective sports category on the development of those who practice it, especially in the sphere of the individual’s personality. In individual sports, the effort, pressure of competition, and actions taken based on the sport’s success or failure belong to the athlete. The athlete is aware of this aspect, he is prepared from a mental point of view, and he is supported and motivated in this direction by the people close to him (the coach, staff, and family). All participants evaluated in the study had been practicing their respective sports for a long time at the senior level, so there was a notable impact on individuals from a psychosocial perspective. The team sports category characteristics refer to the team as a social group. In team sports, only the leaders stand out, which draws attention to the fact that almost everything the other teammates do is transferred to the team. A part of the individual’s issues are related to the team; the responsibilities and expectations are not always distributed equally among the team members. We believe that these aspects have determined the scores that manifested as the difference in self-esteem levels between the two sports categories. For both categories of athletes, it was observed that the level of self-esteem increased from P1 to P2. This interval marks the beginning of the training period and the beginning of the competition period. The tendency of self-esteem to increase shows that the athletes achieved their goals in basic training; progress was made, thus ensuring a fair, promising, and optimistic mood when starting competitions. The score value reflected an average level of self-esteem (at the upper limit of this standard level).

All these aspects also indicate realism, optimal confidence in one’s strengths, and a competitive experience—a context in which exuberance and depression do not manifest. A particular aspect of the research was highlighted on the social courage scale within the Self-Confidence Test. Although the average level was higher for the individual sports category, no statistically significant differences between the two groups of athletes were reported. The level of social courage had no relevant changes between P1 and P2 for any athlete. It seems that this component has a stable tendency. It seems to be a constant within self-esteem, and it is not affected as the other two aspects are. This value scale was similar for all athletes, with no relation to the specificity of the sport practiced. From our perspective, knowledge of the athlete’s level of self-esteem is an essential factor because it reflects the reliable self-evaluation of each athlete, with both positive and negative aspects. Only an optimal level of self-esteem can provide the necessary emotional background for the athlete. The optimal state is when an individual can train effectively to obtain the relevant sports results. Extreme levels of self-esteem (very high or very low) are harmful [23,24,25]. Although the subjects of our research were at appropriate stages, an aspect revealed by the average values, the identification of the “optimum” is made at the individual level; the calibration is performed on the basis of a detailed and personal analysis, with reference to individual particularities, expectations, possibilities, etc. The comparative approach (individual versus team sports) for self-esteem can bring benefits to the category of team sports as well. For example, if the coaches and staff believe that the team’s self-esteem level is too low and that this could affect the sports performance, there is a possibility to solve this problem by adopting a training strategy and an approach specific to the individual sports. Since it was observed that those in the individual sports category had higher levels of self-esteem, a viable option would be the training known in the literature as individualized training [26,27,28]. It is necessary to take into account all the particularities of this type of training and the positive effects that can be obtained physically, technically, tactically, and psychologically by increasing self-esteem.

Çağlayan and Uçan [29] reported no significant differences in self-esteem scores between athletes and non-athletes based on the Rosenberg Self-Esteem Scale results. The same result was obtained for individual and team sport athletes and non-athletes. No significant differences between male individual and team sports athletes in terms of their abilities to express emotions and self-esteem were reported by Akelaitis and Malinauskas [30]. On the basis of our findings, we reported significant differences between the individual and team athletes in self-esteem levels.

We mention the fact that the phrase “social courage” refers to a set of actions that involves certain risks, which are taken mainly for the benefit of others but also with reference to self for welfare and/or performance in various fields of activity [31,32]. The concept of initiating social contacts represents an individual’s ability to develop social relationships with a great impact on long-term attitudes, behaviors, and perceptions [33].

We believe that self-esteem analysis in two sports categories makes the meanings a little too general, but this limitation may constitute future research ideas for which studies could involve fewer sports disciplines and include more participants. Another limiting aspect is that we did not control other factors that could have influenced self-esteem, positively or negatively, for the interval between P1 and P2. We will try to dedicate future research to these aspects to better understand the factors that might affect the athlete’s self-esteem during specific training. The level of an athlete’s self-esteem is valuable knowledge. The involvement of athletes in training and competition depends on this level, and it determines sports performance.

## 5. Conclusions

There are significant differences in self-esteem levels between individual and team athletes. It was noted that for both categories of athletes, the level of self-esteem increased between the two periods of investigation, which we consider positive, as it reflects the growing satisfaction of athletes in achieving short-term goals. Individual athletes presented higher levels of self-esteem. Self-esteem is an aspect that can be further analyzed, corrected, maintained, and stimulated for optimal performance in sports.

## Figures and Tables

**Table 1:** Results obtained for Rosenberg Self-Esteem Scale.

Sports	Testing Periods	*N*	Mean ± SD	S Error M	*t*	*p*
Individual	P1	40	27.80 ± 3.15	0.99	3.73	0.002
	P2	40	32.20 ± 1.98	0.62		
Team	P1	40	22 ± 3.91	1.24	4.13	0.001
	P2	40	28.40 ± 2.95	0.93		
Individual vs. Team	P1	80	27.80 ± 3.15	0.99	3.64	0.002
			22 ± 3.91	1.24		
Individual vs. Team	P2	80	32.20 ± 1.98	0.62	3.37	0.003
			28.40 ± 2.95	0.93		

P1—period 1; P2—period 2; *N*—number of participants; differences in Rosenberg Self-Esteem Scale between groups and periods are presented as means ± standard deviation. *p* < 0.05—significant difference.

**Table 2:** The results obtained in the Self-Confidence Test.

Scale	Sports	Testing Periods	*N*	Mean ± SD	S Error M	*t*	*p*
Self-confidence	Individual	P1	40	8 ± 1.15	0.36	2.02	0.058
		P2	40	9 ± 1.05	0.33		
	Team	P1	40	6.50 ± 1.17	0.37	2.81	0.010
		P2	40	7.90 ± 0.99	0.31		
	Individual vs. Team	P1	80	8 ± 1.15	0.36	2.87	0.010
				6.50 ± 1.17	0.37		
	Individual vs. Team	P2	80	9 ± 1.05	0.33	2.40	0.027
				7.90 ± 0.99	0.31		
Social courage	Individual	P1	40	7.70 ± 1.63	0.58	0.16	0.879
		P2	40	7.80 ± 1.22	0.38		
	Team	P1	40	7.10 ± 1.10	0.35	0.44	0.669
		P2	40	7.30 ± 0.94	0.30		
	Individual vs. Team	P1	80	7.70 ± 1.63	0.58	0.92	0.349
				7.10 ± 1.10	0.35		
	Individual vs. Team	P2	80	7.80 ± 1.22	0.38	1.02	0.322
				7.30 ± 0.94	0.30		
Initiation of social contacts	Individual	P1	40	8 ± 1.15	0.36	1.96	0.065
		P2	40	8.90 ± 0.87	0.27		
	Team	P1	40	6 ± 1.88	0.59	1.42	0.171
		P2	40	7.20 ± 1.87	0.69		
	Individual vs. Team	P1	80	8 ± 1.15	0.36	2.86	0.010
				6 ± 1.88	0.59		
	Individual vs. Team	P2	80	8.90 ± 0.87	0.27	2.60	0.018
				7.20 ± 1.87	0.69		

P1—period 1; P2—period 2; *N*—number of participants; differences in Rosenberg Self-Esteem Scale between groups and periods are presented as mean ± standard deviation. *p* < 0.05—significant difference.
